# Toxic Epidermal Necrolysis associated with COVID‐19 infection: A case report

**DOI:** 10.1002/ccr3.5565

**Published:** 2022-03-13

**Authors:** Lamia Jouhar, Marva Yahya, Sohair Elsiddiq

**Affiliations:** ^1^ 36977 Department of Pediatrics Hamad Medical Corporation Doha Qatar

**Keywords:** COVID‐19, dermatology, pediatrics, toxic epidermal necrolysis

## Abstract

Toxic Epidermal Necrolysis/Steven–Johnson Syndrome (TEN/SJS) is one of the most serious dermatological adverse reactions triggered mainly by drugs and less likely by infections. COVID‐19 disease is caused by Sever Acute Respiratory Syndrome Coronavirus 2 (SARS‐CoV‐2) with a wide range of clinical manifestations. Skin involvement is common in COVID‐19 patients including urticaria, purpura, and vasculitis. There were reported cases of TEN/SJS in adults with COVID‐19 infections and only two reported cases in pediatric patients. The causality relationship between COVID‐19 infection and TEN/SJS was not established in most cases due to history of drug usage that could be the trigger. In this study, we are reporting a case of previously healthy child apart from COVID‐19 infection who was admitted to the intensive care unit with TEN involving more than 30% of body surface area confirmed by skin biopsy. The child was treated with intravenous immunoglobulins, steroids, and cyclosporin with a very good outcome.

## Graphical abstract

Whether COVID‐19 infection can trigger TEN in pediatrics patient is an important question that needs larger studies, yet it is worth reporting this case of possible correlation between COVID‐19 and TEN in pediatrics.

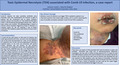

## 1 INTRODUCTION

COVID‐19 infection has been spreading worldwide since December 2019.^1^ Skin manifestations are common as 60% of patients had skin involvement such as rashes, urticaria, purpura, and vasculitis.^2^ Toxic Epidermal Necrolysis (TEN) is a life‐threatening dermatological disease distinguished from Steven–Johnson syndrome (SJS) by the percentage of body surface area involved (>30% for TEN, <10% for SJS). The pathophysiology is not fully understood yet the disease is linked to immune system activation triggered by drugs or infections or unknown causes. TEN/SJS presents with erythematous macules that develop central necrosis and bullae lesions followed by a painful full‐thickness skin and mucosal membranes exfoliation. The diagnosis is made based on clinical features in addition to histopathology findings.^3^ The mainstay of treatment is supportive care and local wound care. Studies in children showed better mortality and morbidity outcomes with using Intra Venous Immunoglobulins (IVIG) and steroids. Other agents can be used such as cyclosporin, plasmapheresis, and TNF a inhibitors.^4^ In this study, we are reporting a case of biopsy‐confirmed TEN in pediatrics patient with a history of recent COVID‐19 infection. Informed consent was obtained from the parents and the letter was approved by the Institutional Review Board (IRB) at out institution.

## 2 CASE DESCRIPTION

**FIGURE 1:**
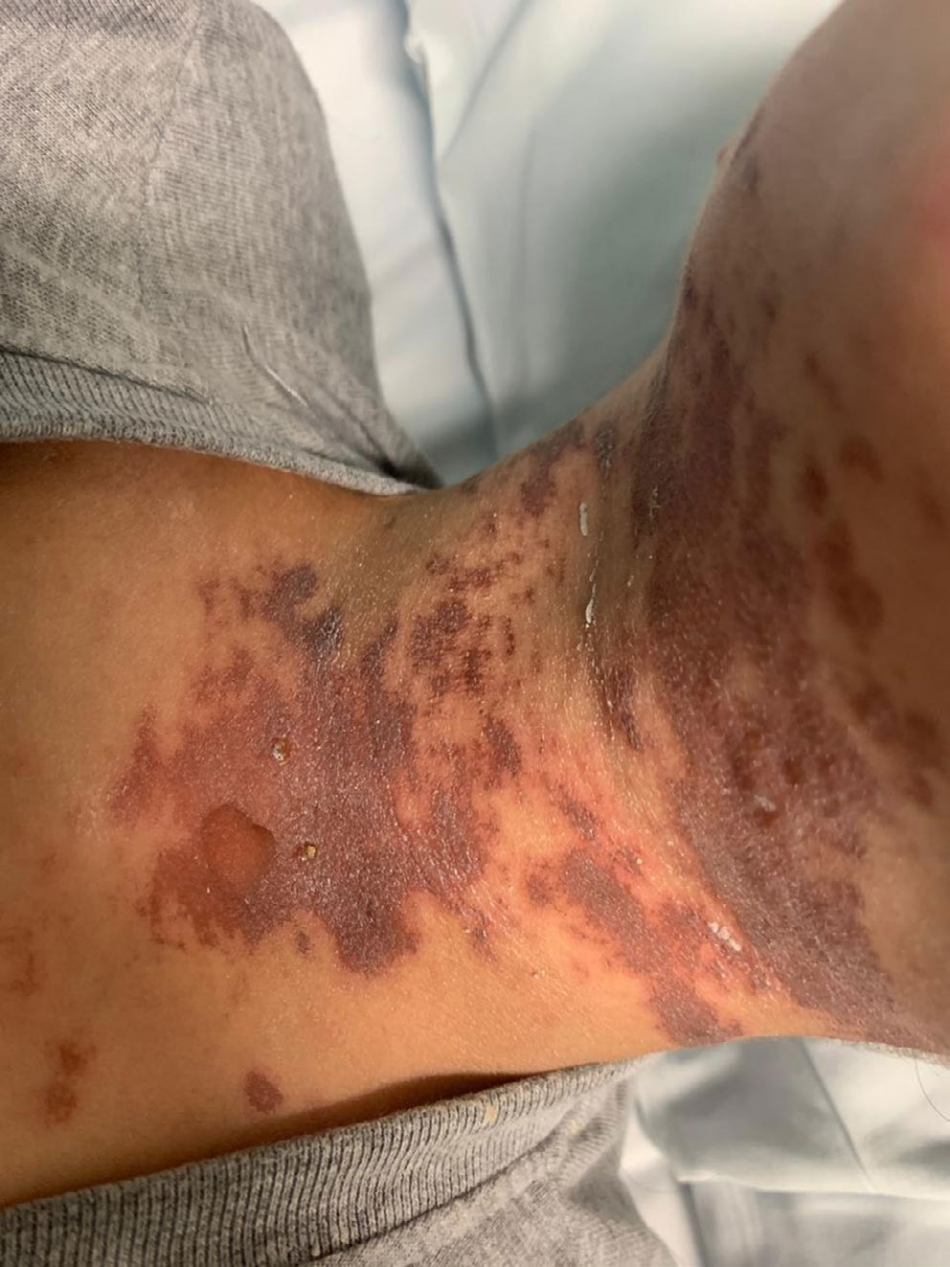
Skin rash on the neck

**FIGURE 2:**
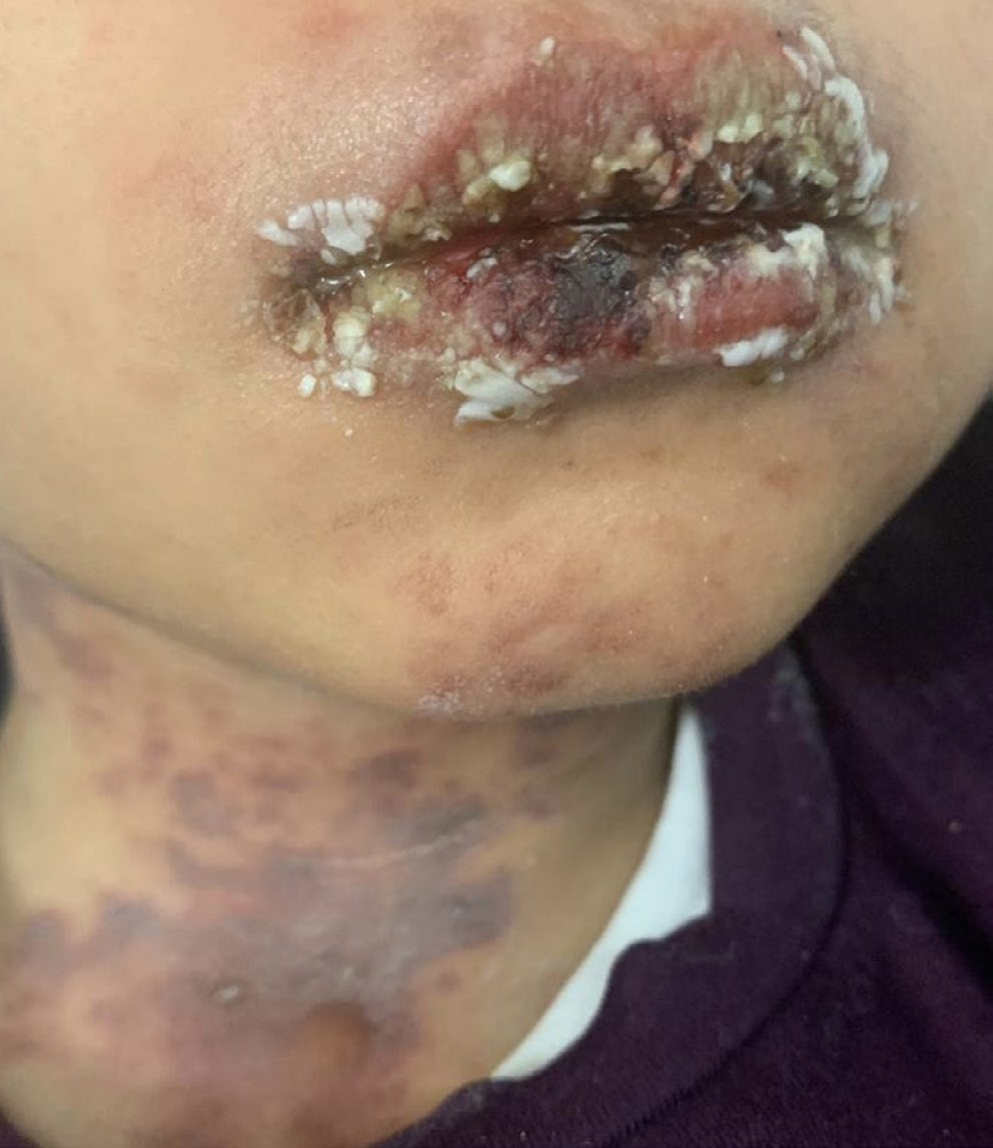
Mucus membranes involvement

A 6‐year‐old previously healthy boy presented with fever, oral ulcers, and maculopapular rash on the trunk and extremities involving the palms and soles for 2 days. He was tested for COVID‐19 infection as a screening due to contact with positive family member 2 weeks prior to this presentation and had mild symptoms treated supportively at home. He had a history of Ibuprofen use 2 weeks prior to presentation. He was admitted to the hospital for supportive care. During his hospitalization, he had low grade fever and was hemodynamically stable. His skin rash was progressing to violaceous targetoid lesions on the trunk and extremities with genital erosion, Nickolsky sign was positive (Figure 1). He had mild purulent conjunctivitis and crusted lips lesions with de‐epithelization of the oral mucosa (Figure 2). He was seen by dermatologist and the clinical picture was consistent with TEN. SCROTEN score was 2 for detachment more than 30%, low bicarbonate of 19. Laboratory workup showed a normal complete blood count (CBC), elevated CRP 40 mg/dl, normal renal and liver function tests. Viral PCR in blood (adenovirus, EBV, CMV, parvovirus, HHV6, measles, enterovirus) was negative. Respiratory viruses PCR including Mycoplasma PCR in nasopharyngeal swab was negative. COVID‐19 PCR was positive 10 days back. Blood culture showed no growth and eye culture showed methicillin‐sensitive Staph aureus (MSSA). A 4‐mm puh skin biopsy from target necrotic detached skin showed sloughed necrotic epidermis. Necrotic keratinocytes were abundant, the superficial dermis showed marked perivascular and interstitial lymphocytic inflammatory infiltrates. The histological features were consistent with SJS/TEN. Multi‐system Inflammatory Syndrome related to COVID‐19 infection (MIS‐C) was on the differential diagnosis, but the child had only skin and mucous membranes involvement with no respiratory, gastroenterology, or other systems symptoms. Laboratory workup was not supportive of MIS‐C too with normal white blood cells (WBC), Lymphocyte's count, ferritin 102 µg/L, and IL6: 42 pg/ml. He was treated with supportive care, IVIG 1 g/Kg daily for 5 days, IV dexamethasone shifted later to oral Prednisolone, cyclosporin 3 mg/kg/day. His rash and oral mucositis improved within 1 week, and he was discharged in stable condition. He was seen in the clinic after 1 month of discharge and recovery of the skin, oral and eye mucosa was observed.

## 3 DISCUSSION

This report presented a case of a child with TEN and history of recent COVID‐19 infection. Most cases of TEN/SJS are triggered by drugs mainly sulfonamides and anticonvulsants. It usually occurs 4–28 days after drug exposure, delineating the importance of obtaining a detailed drug exposure history.^5^ Some cases were reported with infections such as mycoplasma pneumoniae, viruses (Coxsakie, influenza, Epstein–Barr, human herpes virus 6 and 7, cytomegalovirus, parvovirus), bacterial infections such as (Streptococcus group A) and mycobacterium.^4^

There were case reports of TEN/SJS associated with COVID‐19 infection in adults with probable association with drugs such as hydroxychloroquine,^5^ Allopurinol/ Septrin/ Lenalidomide,^6^ Lamotrigine,^7^ one case with no history of drug exposure.^8^ A report of more than 5000 pediatrics patients with COVID‐19 infection reported skin manifestations in 0.25% of the cases. Only one patient had SJS with pulmonary involvement and patient deceased.^9^ Another case of an 8‐year‐old boy with COVID‐19 infection and lung involvement who developed SJS rash was reported. The child improved with IVIG, cyclosporin, and steroids treatment.^10^ Both cases reported a history of amoxicillin‐clavulanate use. In our case, the relationship between COVID‐19 infection and TEN is not clear as the child had a history of Ibuprofen use that could be the culprit trigger. However, COVID‐19 could still be the trigger in this case. It is worth reporting this case to keep in mind the wide spectrum of dermatological presentation in COVID‐19 patients.

## CONFLICT OF INTEREST

The authors have no conflict of interest to disclose.

## AUTHOR CONTRIBUTIONS

Lamia Jouhar involved in literature review, writing the manuscript, and obtaining the consent. Marva Yahya and Sohair Elsiddiq involved in reviewing the manuscript.

## ETHICAL APPROVAL

Informed consent was obtained from the parents. The case was approved by the Institutional Review Board at Hamad Medical Corporation.

## CONSENT

Written informed consent was obtained from the parents.

## Data Availability

Data sharing not applicable to this article as no datasets were generated or analyzed during the current study.
